# Impact of rotavirus vaccination on rotavirus genotype distribution and diversity in England, September 2006 to August 2016

**DOI:** 10.2807/1560-7917.ES.2019.24.6.1700774

**Published:** 2019-02-07

**Authors:** Daniel Hungerford, David J Allen, Sameena Nawaz, Sarah Collins, Shamez Ladhani, Roberto Vivancos, Miren Iturriza-Gómara

**Affiliations:** 1 The Centre for Global Vaccine Research, Institute of Infection and Global Health, University of Liverpool, Liverpool, United Kingdom; 2 Field Epidemiology Services, National Infection Service, Public Health England, Liverpool, United Kingdom; 3 NIHR Health Protection Research Unit in Gastrointestinal Infections, Liverpool, United Kingdom; 4 NIHR Health Protection Research Unit in Emerging and Zoonotic Infections, Liverpool, United Kingdom; 5 Department of Pathogen Molecular Biology, Faculty of Infectious and Tropical Diseases, London School of Hygiene and Tropical Medicine, London, United Kingdom; 6 Virus Reference Department, National Infection Service, Public Health England, London, United Kingdom; 7 Immunisation Department, National Infection Service, Public Health England, London, United Kingdom; 8 NIHR Health Protection Research Unit in Immunisation, London School of Hygiene and Tropical Medicine, London, United Kingdom

**Keywords:** vaccine-preventable diseases, rotavirus, molecular methods, surveillance, epidemiology, typing

## Abstract

**Introduction:**

Rotavirus vaccination with the live-attenuated monovalent (a G1P[8] human rotavirus strain) two-dose Rotarix vaccine was introduced in England in July 2013. Since then, there have been significant reductions in rotavirus gastroenteritis incidence.

**Aim:**

We assessed the vaccine’s impact on rotavirus genotype distribution and diversity 3 years post-vaccine introduction.

**Methods:**

Epidemiological and microbiological data on genotyped rotavirus-positive samples between September 2006 and August 2016 were supplied by EuroRotaNet and Public Health England. Multinomial multivariable logistic regression adjusting for year, season and age was used to quantify changes in genotype prevalence in the vaccine period. Genotype diversity was measured using the Shannon’s index (H′) and Simpson’s index of diversity (D).

**Results:**

We analysed genotypes from 8,044 faecal samples. In the pre-vaccine era, G1P[8] was most prevalent, ranging from 39% (411/1,057) to 74% (527/709) per year. In the vaccine era, G1P[8] prevalence declined each season (35%, 231/654; 12%, 154/1,257; 5%, 34/726) and genotype diversity increased significantly in 6–59 months old children (H’ p < 0.001: D p < 0.001). In multinomial analysis, G2P[4] (adjusted multinomial odds ratio (aMOR): 9.51; 95% confidence interval (CI): 7.02–12.90), G3P[8] (aMOR: 2.83; 95% CI: 2.17–3.81), G12P[8] (aMOR: 2.46; 95% CI: 1.62–3.73) and G4P[8] (aMOR: 1.42; 95% CI: 1.02–1.96) significantly increased relative to G1P[8].

**Conclusions:**

In the context of reduced rotavirus disease incidence, genotype diversity has increased, with a relative change in the dominant genotype from G1P[8] to G2P[4] after vaccine introduction. These changes will need continued surveillance as the number and age of vaccinated birth cohorts increase in the future.

## Introduction

Prior to the introduction of rotavirus vaccination, Group A rotaviruses were the most common cause of severe childhood diarrhoea globally, resulting in over 450,000 deaths in children aged under 5 years [1]. In England and Wales, rotavirus accounted for ca 80,000 general practice (GP) consultations and was responsible for 45% of acute gastroenteritis hospital admissions [2,3]. In July 2013, the United Kingdom (UK) introduced the monovalent (G1P[8]) live-attenuated, two-dose oral human vaccine (Rotarix, GlaxoSmithKline Biologicals, Rixensart, Belgium) into the routine childhood immunisation schedule at 8 and 12 weeks of age [4]. Rotavirus vaccine uptake increased rapidly to over 90% for one dose and has remained consistently high; in July 2016, vaccine uptake was over 94% for one dose and over 90% for the recommended two-dose schedule [5]. In high-income countries, efficacy of the monovalent G1P[8] Rotarix vaccine against severe rotavirus gastroenteritis was estimated at over 85% and further trials have shown that it is efficacious against multiple rotavirus strains [6,7]. Since vaccine implementation in the UK, data from laboratory reports, hospital admissions and GP consultations have shown a significant reduction in the incidence of rotavirus gastroenteritis in vaccine eligible children (children born since 01 May 2013), older vaccine ineligible children and adults (≥ 18 years) [8-11].

Group A rotaviruses are defined by the middle capsid antigen and further classified into G and P types based on serological and genetic characterisation of two outer viral proteins, the VP7 (a glycoprotein) and the VP4 (a protease sensitive protein), respectively. Furthermore, whole genome sequencing has allowed rotavirus strains to be classified according to genotype constellations by using a common genetic backbone constellation (defined by nine of eleven gene segments, excluding VP7 and VP4). Among human group A rotaviruses there are two common genotype constellations: Wa-like and DS-1 like [12].

Prior to introduction of rotavirus vaccination, genotype G1P[8] was the predominant circulating rotavirus genotype in the UK [13-17]. In England, like in most countries that have recently introduced rotavirus vaccination, monitoring the impact of the rotavirus vaccine includes the assessment of changes in healthcare utilisation and disease incidence, as well as monitoring genotype distribution. Rotavirus genotype surveillance has been undertaken systematically pre- and post-vaccine introduction in the context of the European Rotavirus Network, EuroRotaNet, which was established in January 2007 and has 14 member countries, including the UK [18,19]. Each member country conducts rotavirus genotype surveillance; laboratory and epidemiological data are also collated within the network to generate comprehensive information on the rotavirus genotypes co-circulating throughout Europe. This multi-centre and data-sharing network approach allows widespread monitoring of circulating rotavirus genotypes in order to identify: (i) possible vaccine-induced emergence of antibody escape mutants; (ii) possible emergence of non-vaccine genotypes; and (iii) possible emergence of reassortants between vaccine-type and naturally circulating wild-type strains.

Because England has one of the largest historical rotavirus genotype databases in Europe, dating back to the late 1990s [13,16,17], and has used consistent laboratory diagnostics and genotyping methods, it is an ideal study location for monitoring any changes in genotype distribution post-vaccine introduction. This study evaluates the impact of routine childhood rotavirus vaccination on relative changes in rotavirus genotype distribution and diversity in England.

## Methods

### Study area and samples

The study area was England, covering all regions. Public Health England (PHE), Colindale, receives electronic laboratory reports of laboratory-confirmed rotavirus infections from diagnostic laboratories in England and Wales. In this study, samples included rotavirus-positive faecal samples from mostly sporadic gastroenteritis cases (if associated with outbreaks, only a single sample per outbreak) submitted for routine diagnostic testing and genotyped at the PHE Virus Reference Department (VRD) using standardised G and P typing methods [14,20]. 

Prior to vaccine introduction, selection of rotavirus-positive samples for genotyping was passive. As part of EuroRotaNet, regional laboratories in England submitted any rotavirus-positive residual samples to the VRD. Although submission of samples to VRD was not actively followed-up, at a country level the minimum target number of specimens included for genotyping would enable detection of rotavirus genotypes with a prevalence of ≥ 1% [19]. 

When the infant rotavirus vaccination programme was introduced in July 2013, the Immunisation Department at PHE initiated active surveillance for rotavirus. Hospital laboratories across England and Wales were actively requested to submit all rotavirus-positive samples in vaccine-eligible children to the PHE VRD for confirmation and genotyping. After the introduction of rotavirus vaccination G1P[8] vaccine-derived strains were defined on the basis of the sequences of the VP4 and VP7 encoding genes displaying highest homology with Rotarix sequences and/or through the detection of the Rotarix strain using a published and validated qRT-PCR assay which specifically targets the non-structural protein 2 (NSP2) gene of the Rotarix strain [21].

### Data

The sampling methods used mean that the data in this study do not represent rotavirus incidence but allow us to assess relative changes in genotype distributions. Details on case age, region, specimen collection date and rotavirus genotyping results for specimens collected between September 2006 and August 2016, were included in this study. Data on cases’ sex were incomplete, so we were unable to include these in the analyses however, previous analysis of EuroRotaNet data has shown that there is no difference in genotype distribution by sex [19]. 

While EuroRotaNet was established in January 2007, data dating back to September 2006 were retrospectively collected by this network. Thus data from September 2006 to December 2012 are held by EuroRotaNet and, and from January 2013 to August 2016, jointly by EuroRotaNet and PHE. In order to maintain complete surveillance years (September to August) for analysis, the pre-vaccine period was defined as September 2006 to August 2013 and the vaccine period was defined as September 2013 to August 2016.

Age groups of cases (< 6 months, 6–11 months, 12–23 months, 2–4 years and ≥  5 years) were constructed using date of birth and date of specimen submission. Genotyped rotavirus strains were categorised according to their possible evolutionary origin [14]. A derived binary ‘season’ variable was constructed to indicate whether a rotavirus case occurred during the pre-vaccine introduction peak rotavirus season (calendar week 1 to week 25) or the non-peak rotavirus season (calendar week 26 to week 52) according to the date of specimen collection [17].

For the purposes of statistical analyses (apart from genotype diversity), because of the large number of genotypes, any single genotype which contributed < 1% of samples over the study period was classified into a generic ‘other’ category for ‘rarer’ genotypes. A separate grouping ‘mixed or untypable’ was established for any mixed (more than one genotype found in one individual sample) or partially typed strains.

### Vaccination status

As part of the enhanced surveillance, the general practitioner of each rotavirus-positive infant in the vaccine-eligible age group (born since 01 May 2013) was contacted to establish their rotavirus vaccine history. For this study, the vaccine history was assessed for samples with a G1P[8] vaccine-derived strain. The vaccination history, included number of rotavirus vaccine doses received and date of vaccination.

### Data analysis

#### Descriptive analysis

Data analyses were performed using R version 3.3.2 [22]. The distribution of rotavirus G- and P-genotypes was tabulated in respect to age group, year, and period (pre- or post-vaccine introduction). Differences between continuous variables were tested using Student’s t-test or Wilcoxon rank-sum test if not normally distributed and chi-squared-test or Fisher’s exact test for categorical variables. Any G1P[8] vaccine-derived samples were excluded from the statistical analyses under the assumption that they were not the disease causative agent.

#### Genotype differences pre- and post-vaccine introduction

To investigate relative statistical differences in circulating genotypes pre- and post-vaccine introduction, we fitted multinomial logistic regression models, with genotype as the outcome and G1P[8] as the baseline genotype. Model fitting was based on season, surveillance year and age group; these terms were identified a priori [17]. The model was first run as a univariable analysis including only the binary vaccine period variable for before or after vaccine introduction. Age-stratified models were also run; with genotype as the outcome variable, and vaccine period, season and surveillance year as covariates. Multinomial odds ratios (MOR), 95% confidence intervals (CI) and the associated p values were calculated from the Wald test. Results were considered significant at p < 0.05. 

Rotavirus genotype diversity was compared using two established biodiversity indices, Simpson’s index of diversity (D) and Shannon’s index (H) [23]. All single typed rotavirus genotypes were included in the analysis. Simpson’s index of diversity (D) has a range between 0 and 1 (maximum diversity) and represents the probability that two randomly chosen rotavirus genotypes will have different G and P types and is calculated as 1  −  λ, where λ = Σ(pi2) and pi is the proportional abundance of a genotype i. Shannon’s index (H′) ranges from 0 (no diversity) to Y (maximum) and quantifies the uncertainty in predicting the rotavirus genotype identity of an individual sample that is taken at random from the dataset and is calculated as H′ =   − Σ(pi  x  ln(pi)) [17]. Confidence intervals were estimated using bootstrap resampling methodology and differences by age group, pre- and post-vaccine introduction were compared using p values generated from z-scores during bootstrap analysis. The R packages ‘vegan’ and ‘boot’ were used for the analysis of genotype biodiversity [24,25].

## Results

### Descriptive statistics

**Figure 1:**
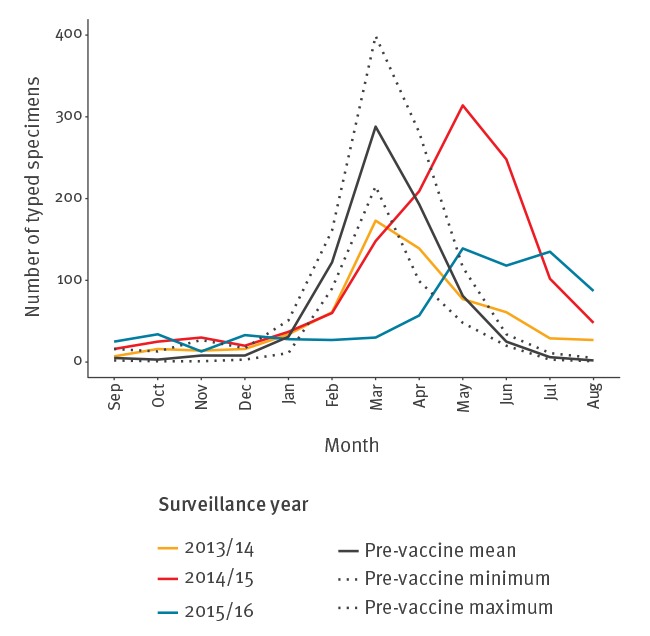
Number of rotavirus specimens typed per month and surveillance year, England, September 2006–August 2016 (n = 8,044)

In England a total of 8,044 rotavirus-positive specimens were genotyped between September 2006 and August 2016. Six per cent of specimens (n = 466) had no detail on case age, and most of these were from 2007/08 (n = 209/737; 28%) and 2011/12 (n = 137/732; 19%). The case age ranged from <1 month to 104 years and the majority of specimens were from children aged < 5 years (92%; 6,945/7,578), with most specimens (56%; 4,237/7,578) from children aged 6–23 months (Table). Prior to vaccine introduction, the rotavirus season consistently occurred between January and May, with the peak occurring during March. After the introduction of rotavirus vaccination, the seasons became less pronounced; in the first surveillance year post-vaccine introduction (2013/14), the peak occurred in March, but in 2014/15 and 2016/17 the peak was in May and season lasted longer into July (Figure 1).

**Table:** Number of rotavirus specimens collected in the pre-vaccine era and the vaccine era, by genotype and age group, England, September 2006–August 2016 (n = 8,044)

Characteristic	Pre-vaccine era		Vaccine era							
	Sep 2006–Aug 2013 (n = 5,407)		Sep 2013–Aug 2014 (n = 654)		Sep 2014–Aug 2015 (n = 1,257)		Sep 2015–Aug 2016 (n = 726)		Sep 2013–Aug 2016 (n = 2,637)	
**Genotype**	**n**	**%**	**n**	**%**	**n**	**%**	**n**	**%**	**n**	**%**
G1P[8]	2,987	55	231	35	154	12	34	5	419	16
G2P[4]	397	7	28	4	345	27	325	45	698	26
G3P[8]	527	10	190	29	101	8	61	8	352	14
G4P[8]	368	7	53	8	103	8	21	3	177	7
G8P[4]	118	2	0	0	0	0	2	0	2	0
G9P[8]	597	11	46	7	191	15	134	18	371	14
G12P[8]	122	2	19	3	242	19	17	2	278	11
G1P[8]-unknown^a^	0	0	0	0	0	0	3	0	3	0
G1P[8]-VD	0	0	64	10	84	7	67	9	215	8
Other^b^	91	2	6	1	27	2	32	4	65	2
Mixed^c^ and untypable	200	4	17	3	10	1	30	4	57	2
**Age group^d^**	**n**	**%**	**n**	**%**	**n**	**%**	**n**	**%**	**n**	**%**
** **< 6 months	577	11	169	25	233	19	102	15	504	20
6–11 months	1,194	24	105	16	124	9	61	9	290	11
12–23 months	1,851	37	261	40	407	34	234	34	902	35
2–4 years	940	19	81	12	369	34	238	35	688	27
** **≥ 5 years	464	9	37	6	79	7	52	8	168	7

VD: vaccine-derived.

^a^ G1P[8]-unknown: unknown whether these strains are wild-type G1P[8] or VD.

^b^ ‘Other’ refers to rarer genotypes, which contributed < 1% of samples over the study period.

^c^ Mixed: more than one genotype found in an individual sample.

^d ^There were 466 specimens with unknown case age. Percentages are calculated from total specimens with known case age.

Figure is not representative of rotavirus disease incidence.

### Genotype distribution

**Figure 2:**
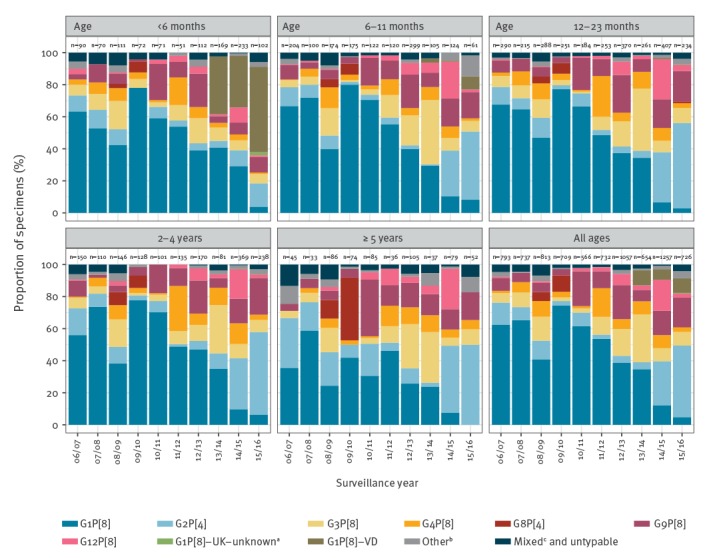
Genotype proportions of typed rotavirus specimens by surveillance year and age group, England, September 2006–August 2016 (n = 8,044)

UK: United Kingdom; VD: vaccine derived.

^a^ G1P[8]-unknown: unknown whether these strains are wild-type G1P[8] or VD.

^b^ ‘Other’ refers to rarer genotypes which contributed < 1% of samples over the study period.

^c^ Mixed: more than one genotype found in an individual sample.

In the pre-vaccine period (September 2006 to August 2013), G1P[8] was the overall predominant genotype (55%), ranging from 39% (411/1,057) to 74% (527/709) per year (Table). In cases aged 5 years and older, G1P[8] was less dominant than in younger age groups, representing 34% (156/464) of all specimens genotyped, while genotypes G2P[4] (16%; 73/464) and G8P[4] (8%; 39/464) were more prevalent (Figure 2).

There were 466 specimens with unknown case age. Percentages are calculated from total specimens with known case age. The genotyping analyses’ results are indicated by different colours, as explained in the legend.

In the first surveillance year of the vaccine era (2013/14), G1P[8]-type viruses appeared to become less dominant overall, accounting for 35% of genotyped samples; in the same period, G3P[8] accounted for 29% (Table). In surveillance years two (2014/15) and three (2015/16) of the vaccine era, G2P[4]-type viruses accounted for the highest proportion of strains (27% and 45%, respectively). In these two surveillance years wild-type G1P[8] viruses seemed to become less prevalent, accounting for 12% of genotyped specimens in 2014/15 and 5% in 2015/16. Also, in 2014/15, G12P[8]-type viruses represented 19% of samples, compared with 2% in the pre-vaccine period.

In the pre-vaccine period G1P[8] was dominant in all children under 5 years of age (Figure 2). In the vaccine period, the distribution of rotavirus genotypes in infants younger than 6 months of age was different to the other age groups; wild-type G1P[8] viruses were present in 28% (141/504) of cases and vaccine-derived G1P[8] strains were detected in 38% (193/504) of cases (Figure2). G1P[8] was detected less frequently as age increased, it was detected in 18% (51/290) of 6–11 month olds, 14% (126/902) of 12–23 month olds, 11% (78/688) of 2–4 year olds, and 9% (15/168) of ≥ 5 year-olds. The change in strain distribution between the pre-vaccine and vaccine era for all ages was significant when excluding vaccine-derived G1P[8] strains (chi-squared = 1,509, degrees of freedom = 8; p < 0.001).

### Vaccine-derived strains

After rotavirus vaccine introduction in the UK in July 2013 there were 215 G1P[8] vaccine-derived strains detected. 

Of the 208 with an age recorded, 92% (n = 191/208) were detected in infants aged 2–5 months of age, who would have been eligible for immunisation. Among these cases, six were unvaccinated infants, vaccine status was unknown for three and five were vaccinated after the sample collection date. The remaining (n = 177) were vaccinated with a median time between specimen date and most recent rotavirus vaccine dose of 13 days (interquartile range (IQR): 7–23) and a maximum of 154 days.

There were 12 instances where vaccine-derived G1P[8] strains were found in specimens from children eligible for vaccination but older than 6 months of age when the sample was taken. Of these, one sample had come from an unvaccinated patient and one from a non-UK resident; the remaining 10 were from patients who had received at least one dose of rotavirus vaccine between 83 days and 420 days before the sample collection date. 

A further two cases were aged < 2 months of age; one had received the first dose of rotavirus vaccine 5 days before the date of sample collection and the other case had not received any vaccine doses at the time of sampling.

Three cases, aged 23 months, 3 years and 4 years were born before vaccine introduction and therefore, would have been too old for the UK rotavirus immunisation programme. Vaccine status for a 23 month-old and 4 year-old could not be followed-up, but the 3 year-old had received the second dose of the rotavirus vaccine 2 days before the date of the sample.

### Age

In the pre-vaccine period, the majority of samples referred were from children aged 6–23 months (61%; 3,045/5,026). In the vaccine era, when excluding G1P[8] vaccine-derived cases, most samples were from children aged 12–59 months (68%; 1,584/2,344). In the pre-vaccine era, the median age was 13 months (IQR: 9–24) compared with 19 months (IQR: 11–29) in the vaccine period (W = 572,300; p < 0.001).

### Multinomial logistic regression

**Figure 3:**
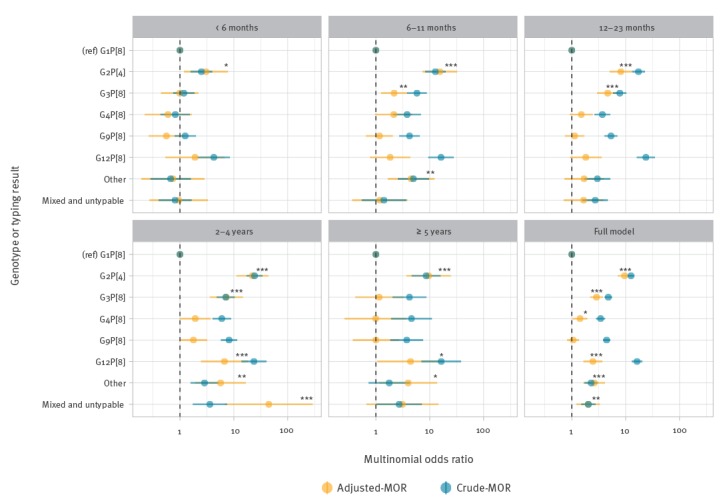
Age-stratified crude and adjusted multinomial odds ratios for genotypes occurring before and after rotavirus vaccine introduction, England, September 2006–August 2016 (full model; n = 7,368)

MOR: multinomial odds ratio; ref: reference.

Although G8P[4] was detected in more than 1% of samples across the study period, for the multinomial regression analyses, G8P[4] was classified as a rare genotype because it was only detected in 2008/09 (n = 45); 2009/10 (n = 73) and 2015/16 (n = 2). We also excluded G1P[8] vaccine-derived strains from the models, under the assumption that the majority were detected in vaccinated infants and were, therefore, not the causative agent of the gastroenteritis symptoms. When adjusting for surveillance year, season and age group, the adjusted multinomial odds ratios (aMOR) of infection caused by the following genotypes: G2P[4] (aMOR: 9.51; 95% CI: 7.02–12.90; p < 0.001); G3P[8] (aMOR: 2.83; 95% CI: 2.17–3.81; p < 0.001); G12P[8] (aMOR: 2.46; 95% CI: 1.62–3.73; p < 0.001); mixed and untypable (aMOR: 2.00; 95% CI: 1.20–3.36; p < 0.01); and other rarer (aMOR: 2.6; 95% CI: 1.63–4.17; p < 0.001); G4P[8] (aMOR: 1.42; 95% CI: 1.02–1.96; p = 0.03), were higher in the vaccine era relative to wild-type G1P[8], while cases due to G9P[8] (aMOR: 1.06; 95% CI: 0.81–1.38; p = 0.661) were unchanged (Figure 3; full model). In age-stratified analysis, across all age groups G2P[4] became significantly more prevalent in the vaccine era relative to wild-type G1P[8] (Figure 3). In infants younger than 6 months of age, G2P[4] (aMOR: 3.04; 95% CI: 1.18–7.88; p = 0.02) was the only genotype that was significantly more prevalent in the vaccine era relative to wild-type G1P[8].

MORs for vaccine era occurrence were estimated with multinomial logistic regression with the outcome variable genotype (G1P[8] as the reference group). Models were additionally adjusted for surveillance year (September–August) and season (and age group for full model). Specimens with unknown case age (n = 466) were excluded from the analysis. G1P[8] vaccine-derived strains (n = 208) and G1P[8]-unknown (strains where it was unknown whether they were wild type or vaccine derived: n = 2) were excluded from all models, under the assumption that the majority were detected in infants likely to have been vaccinated and were therefore not causative agent of the gastroenteritis symptoms. P values for adjusted multinomial odds ratios * p < 0.05, ** p < 0.01, ***p < 0.001.

### Genotype diversity

**Figure 4:**
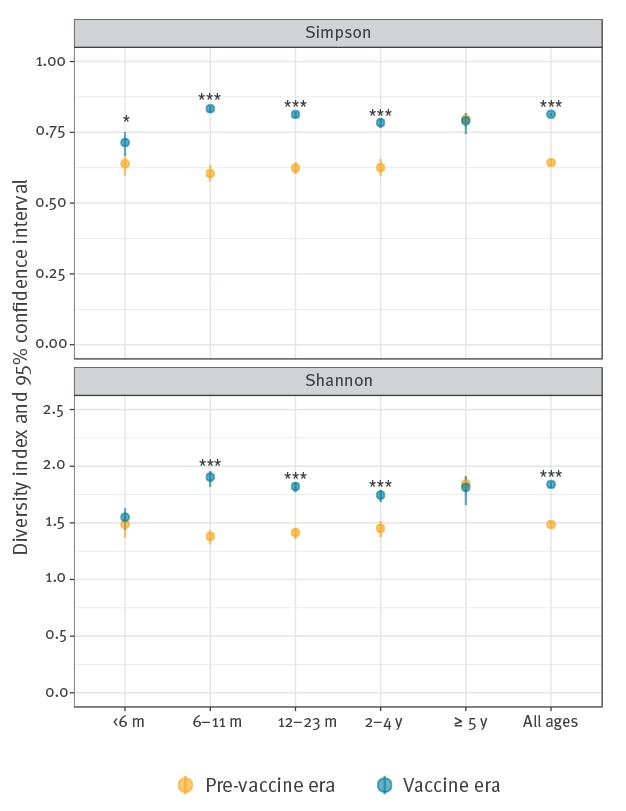
Rotavirus genotype diversity measured using Shannon’s index and Simpson’s index of diversity, with 95% confidence intervals, by age group and vaccine period, England, September 2006–August 2016 (n = 7,128)

There were 30 different single rotavirus genotypes (G10P[4], G12P[10], G12P[4], G12P[6], G12P[8], G1P[4], G1P[5], G1P[6], G1P[8], G2P[10], G2P[4], G2P[6], G2P[8], G2P[9], G3P[14], G3P[3], G3P[4], G3P[6], G3P[8], G3P[9], G4P[4], G4P[8], G6P[14], G6P[9], G8P[14], G8P[4], G8P[8], G9P[4], G9P[6], G9P[8]) identified between September 2006 and August 2016. Genotype diversity increased significantly in the vaccine era compared with the pre-vaccine era (Figure 4), and variation across the age groups was observed. There were significant increases in the genotype diversity among infants aged 6–11 months (H’: p < 0.001; D: p < 0.001); children aged 12–23 months (H’: p < 0.001; D: p < 0.001) and children 24–59 months of age (H’: p < 0.001; D: p < 0.001). However, there was no change in genotype diversity among cases aged 5 years and older (H’: p = 0.477; D: p = 0.471). An increase in genotype diversity was only observed in infants < 6 months of age when using the Simpsons index (D: p = 0.024).

m: months, y: years.

A total of 466 specimens with unknown patient age and 240 mixed or untypable specimens were not considered, as only specimens with a recorded age and single typed rotavirus genotypes are included (G10P[4], G12P[10], G12P[4], G12P[6], G12P[8], G1P[4], G1P[5], G1P[6], G1P[8], G2P[10], G2P[4], G2P[6], G2P[8], G2P[9], G3P[14], G3P[3], G3P[4], G3P[6], G3P[8], G3P[9], G4P[4], G4P[8], G6P[14], G6P[9], G8P[14], G8P[4], G8P[8], G9P[4], G9P[6], G9P[8]). G1P[8] vaccine-derived strains (n = 208) as well as G1P[8]-unknown (i.e. strains where it was unknown whether they were wild-type or vaccine derived; n = 2) were excluded from the diversity analysis, under the assumption that the majority were detected in infants likely to have been vaccinated and were therefore not the causative agent of the gastroenteritis symptoms. P values for a comparison between pre-vaccine and vaccine periods * p < 0.05, ** p < 0.01, ***p < 0.001.

In the pre-vaccine era, genotype diversity was significantly higher in individuals aged 5 years and older than in any other age group but this is no longer true after rotavirus vaccine introduction, genotype diversity was comparable across those aged 6–11 months; 12–23 months, 24–59 months and those aged 5 years and older. Furthermore, genotype diversity in all of these groups was significantly higher than that in infants aged less than 6 months (for all comparison ages, H’: p<0.001 and D’: p<0.001).

## Discussion

There has been a change in rotavirus genotype distribution and diversity in England following the introduction of rotavirus vaccination for all infants in 2013. In the context of very high rotavirus vaccine uptake (ca 94%) [5], there has been both an increase in the genotype diversity among symptomatic cases and a change in the dominant circulating genotype from G1P[8] in the pre-vaccine era to G2P[4] in the vaccine era.

G2P[4] genotypes have been associated with outbreaks in older children (11–12 years) and adults in the absence of rotavirus vaccination or shortly after the introduction of rotavirus vaccination programmes [26-29]. The higher proportion of G2P[4] cases in the vaccine-era has coincided with an increase in the median age of cases, and a decline in cases among vaccine age-eligible age groups. These data may be interpreted in the context of varying strain fitness and partial heterotypic protection. Rotavirus G2P[4] genotypes may be displaced by other, better adapted or fitter genotypes such as G1P[8], particularly in an immunologically naive population. The association of G2P[4] with more frequent infection in older unvaccinated individuals who are likely to have had previous rotavirus infections suggests that the level of cross-protection against G2P[4] is not the same as that against the other genotype constellation 1 (Wa-like) strains (typically G1P[8], G3P[8], G4P[8], G9P[8] and G12P[8]) [29].

In the vaccine era, the higher proportion of wild-type G1P[8] in infants < 6 months of age compared with older age groups could potentially indicate that wild-type G1P[8] remains the most competitive strain when infecting immunologically naive populations. In the absence of a vaccine programme the competitive advantage of G1P[8] strains appears to wane as infants become older and are exposed to repeated infections conferring protection through a varying degree of heterotypic immunity. The introduction of the monovalent rotavirus vaccine provides universal exposure to G1P[8] rotavirus among infants, with protection that is likely to be higher against homotypic strains than heterotypic ones, such as G2P[4], meaning that natural infection leading to disease is more likely to be caused by such heterotypic strains. This is further evidenced by the increase in rotavirus genotype diversity in samples from children aged 6 to 59 months.

The dominance of wild-type G1P[8] in the pre-vaccine period is also consistent with independent studies conducted in England during the late 1990s and early 2000s [13,15,16]. This adds strength to the evidence from this study that the decline in the relative proportion of wild-type G1P[8] and the relative rise of heterotypic strains such as G2P[4] in the vaccine-era is related to vaccine introduction rather than concurrent natural fluctuation. Furthermore, countries such as Belgium, Germany and Scotland introduced the Rotarix vaccine into their national childhood vaccination schedules in 2006, 2013 and 2013, respectively, and have all since seen an absolute decline in G1P[8], with a rise in the proportion and absolute number of G2P[4] strain detections; Belgium reported slightly lower vaccine effectiveness against G2P[4] and Germany detected proportionally more G2P[4] genotypes in Rotarix-vaccinated children [30-33]. This is also true in lower income countries such as Malawi where point estimates for Rotarix vaccine effectiveness were lower against G2 strains than G1 strains (53% vs 82%) [34].

The relative rise of G12P[8] in 2014/15 across all age groups is likely to reflect natural seasonal fluctuations and global emergence rather than a force of vaccine selection. Belgium also experienced an increase in rotavirus cases with G12P[8] detection in 2014/15 [35]. Rapid increases and subsequent declines of G12P[8] have been seen in other European countries; in 2011/12, G12P[8] was the dominant strain in Spain; with the majority of detections occurring in the Gipuzkoa region of north eastern Spain; however, detections of G12P[8] fell in 2012/13 [36]. There has also been independent introduction of G12 strains into Italy in recent years [37]. The emergence of G12 strains in countries both with and without rotavirus vaccination has been reported globally since 2004 [38-40].

In the pre-vaccine era, the predominant genotype G1P[8] declined with increasing age, intimating differences in homotypic and heterotypic immunity generated by natural infection over a person’s life-course. It is, therefore, possible that the concept of ‘antigenic sin’ is applicable to rotavirus and vaccination. If this is the case, it may be expected that immunity to the homotypic strains may be sustained long-term even if circulation of G1P[8] rotaviruses is significantly reduced, through reinfections with other genotypes. This combination of factors poses the question as to whether vaccination will effectively mimic long-term protection previously generated through multiple natural infections and whether this could, in the future, result in a shift to milder disease caused by heterotypic strains in older vaccinated children.

In this study, we identified a number of cases with G1P[8] vaccine-derived strains. The majority of these were detected in vaccinated children aged 2 to 5 months and are likely not to be the cause of the symptoms. These children could be shedding vaccine strain after vaccination and illness is perhaps caused by other infectious pathogens or non-infectious aetiologies [41-43]. Furthermore, the high proportion of G1P[8] vaccine-derived strains may to some extent be the result of the increasing use of molecular methods in diagnostic laboratories across England; these methods will detect shedding of vaccine strain, potentially for months after vaccination, because of higher sensitivity compared with antigen detection methods [44,45].

The detection of G1P[8] vaccine-derived strains in older vaccine-eligible and vaccine-ineligible children would potentially suggest some horizontal transmission from vaccinated infants or persistent shedding in a vaccinated immunosuppressed child, such as those with severe combined immune deficiency [46]. A randomised placebo-controlled trial study in twins showed instances of horizontal transmission of vaccine strains between a vaccinated and unvaccinated twins but without gastroenteritis symptoms [47]. In the case of those G1P[8] vaccine-derived strains detected pre-vaccine introduction, this could be due to: vaccination in another country; privately accessed vaccination; and travel to or contact with children from countries where vaccination was available.

### Strengths and limitations

Our analysis has benefited from England having one of the largest historic rotavirus surveillance systems in Europe. These data provide sufficient sample size year on year to detect genotypes with a prevalence of ≥ 1%, allowing the analyses of the relative genotype prevalence and diversity presented. Importantly, this allowed us to adjust for seasonal trends and fluctuations in the models. We were also able to establish the vaccine status of cases with a detected G1P[8] vaccine-derived strain. 

However, there are limitations which need to be considered. Principally, it is important to be clear that the data included in this study are not representative of disease burden or incidence for the following reasons. Firstly, patients with acute gastroenteritis are advised not to seek medical help, because symptoms are usually self-limiting, particularly in older children and adults. Even if individuals seek medical attention, the National Institute of Clinical Excellent (NICE) guidance and UK guidance advise that testing for rotavirus is a clinical decision, recommended for children under 5 years of age with symptoms, particularly if symptoms are severe, prolonged or atypical [48,49]. Therefore, faecal specimens sent by clinicians for laboratory diagnostics are more likely to be from patients under 5 years of age with moderate to severe disease.

Secondly, even after identifying a rotavirus-positive sample, genotyping is reliant on the laboratory sending the specimen to the PHE VRD. Since 2013, all rotavirus-positive samples from vaccine eligible children have been actively followed up by PHE with the diagnostic laboratory. But, before vaccine introduction in 2013, genotype surveillance of rotavirus-positive samples was more passive and laboratory dependent. Furthermore, the sample size was calculated to detect rotavirus genotypes with a prevalence of ≥ 1% not for estimating rotavirus incidence.

Finally, from 2013 onwards, more laboratories were using molecular methods for diagnostics. As molecular techniques for rotavirus diagnosis are overly sensitive and less representative of symptomatic infection compared with antigen detection methods, it is possible that a greater proportion of samples were from cases where another infectious or non-infectious cause is responsible for the gastroenteritis symptoms. It would, therefore, be beneficial if samples could be tested for other gastrointestinal pathogens, especially in the case of G1P[8] vaccine-derived samples.

## Conclusions

Rotavirus disease burden in England has greatly decreased since the introduction of rotavirus vaccination in 2013. This study shows that rotavirus genotype distribution and diversity has also changed in England since the introduction of vaccination with a large relative shift towards G2P[4], G3P[8] and G12P[8]. These changes will need continued surveillance, especially as the number and age of the vaccinated birth cohort’s increase over the coming years. The results presented here and continued genotype surveillance across Europe through EuroRotaNet will help to inform whether future modifications to rotavirus vaccines may be needed. Ideally, a prospective birth cohort study would be used to assess both disease incidence and prevalence of rotavirus genotypes in symptomatic infections. Specifically, investigating how homotypic and heterotypic protection delivered by the vaccine compares to that induced by natural infection and in relation to age would be relevant.
